# Genetic susceptibility for autoimmune diseases and white blood cell count

**DOI:** 10.1038/s41598-023-32799-8

**Published:** 2023-04-11

**Authors:** Nataraja Sarma Vaitinadin, C. Michael Stein, Jonathan D. Mosley, Vivian K. Kawai

**Affiliations:** 1 Department of Medicine, Vanderbilt University Medical Center, Nashville, TN USA; 2 Department of Pharmacology, Vanderbilt University, Nashville, TN USA; 3 Department of Biomedical Informatics, Vanderbilt University Medical Center, Nashville, TN USA; 4 Division of Clinical Pharmacology, 536 RRB, Vanderbilt University School of Medicine, Nashville, TN 37232 USA

**Keywords:** Genetics, Diseases, Rheumatology, Signs and symptoms

## Abstract

Some autoimmune (AI) conditions affect white blood cell (WBC) counts. Whether a genetic predisposition to AI disease associates with WBC counts in populations expected to have low numbers of AI cases is not known. We developed genetic instruments for 7 AI diseases using genome-wide association study summary statistics. Two-sample inverse variance weighted regression (IVWR) was used to determine associations between each instrument and WBC counts. Effect size represents change in transformed WBC counts per change in log odds-ratio of the disease. For AI diseases with significant associations by IVWR, polygenic risk scores (PRS) were used to test for associations with measured WBC counts in individuals of European ancestry in a community-based (ARIC, n = 8926), and a medical-center derived cohort (BioVU, n = 40,461). The IVWR analyses revealed significant associations between 3 AI diseases and WBC counts: systemic lupus erythematous (Beta = − 0.05 [95% CI, − 0.06, − 0.03]), multiple sclerosis (Beta =  − 0.06 [− 0.10, − 0.03]), and rheumatoid arthritis (Beta = 0.02 [0.01, 0.03]). PRS for these diseases showed associations with measured WBC counts in ARIC and BioVU. Effect sizes tended to be larger among females, consistent with the known higher prevalence of these diseases among this group. This study shows that genetic predisposition to systemic lupus erythematosus, rheumatoid arthritis, and multiple sclerosis was associated with WBC counts, even in populations expected to have very low numbers of disease cases.

## Introduction

Autoimmune (AI) diseases such as systemic lupus erythematous (SLE), rheumatoid arthritis (RA) and multiple sclerosis (MS), are the consequence of an individual’s immune system inappropriately responding to self-tissue^1,2^. While a polygenic predisposition contributes to disease risk for AI diseases^3–5^, AI diseases are relatively uncommon (for instance, crude incidence rates of SLE are 5.3/100,000 person-years in a European ancestry population)^6^. This is due, in part, to the fact that an environmental exposure is often required to trigger the production of disease-facilitating autoantibodies^7^.

The diagnostic criteria for AI diseases typically require the presence of minimal number of stereotypical clinical features and may take many years for an individual to develop a sufficient number of symptoms to meet the diagnostic criteria for an AI disease^8–11^. Thus, the prevalence of individuals with subclinical or variably penetrant disease may be higher than prevalence estimates suggest^12^.

White blood cell (WBC) counts in peripheral blood are a quantitative trait, and increased or decreased counts are part of stereotypical disease process for AI disorders^13,14^. In general, quantitative traits are more sensitive than binary traits (e.g., disease status) to detect associations. Thus, we examined whether polygenic predictors of AI diseases were associated with WBC counts measured in large, unselected populations to test the hypothesis that for relatively rare conditions, such as AI diseases^15^, significant associations can be detected in populations where the disease prevalence would be expected to be low based on estimates from epidemiological studies if the disease is more common than expected. If these associations reflect disease mechanisms, they should follow a pattern consistent with the known epidemiology of the disease, consequently the magnitude of associations would differ among men and women.

We examined several AI diseases: SLE is an uncommon AI disease^16^ with a strong genetic component^3^ that is characterized by decrease in WBC counts in response to disease activity^13,17^; RA is a more common AI disease than SLE^18^ that may exhibit moderate increases in WBC during active disease^14^; and, because there is substantial genetic overlap among AI diseases^4,19,20^, we also included other common AI diseases (ulcerative colitis—UC, Crohn’s disease—CD, MS, Type 1 diabetes—T1D, and autoimmune thyroiditis—AIT). We observed that a genetic predisposition to several AI diseases was associated with WBC counts with larger effects in females.

## Methods

**Figure 1:**
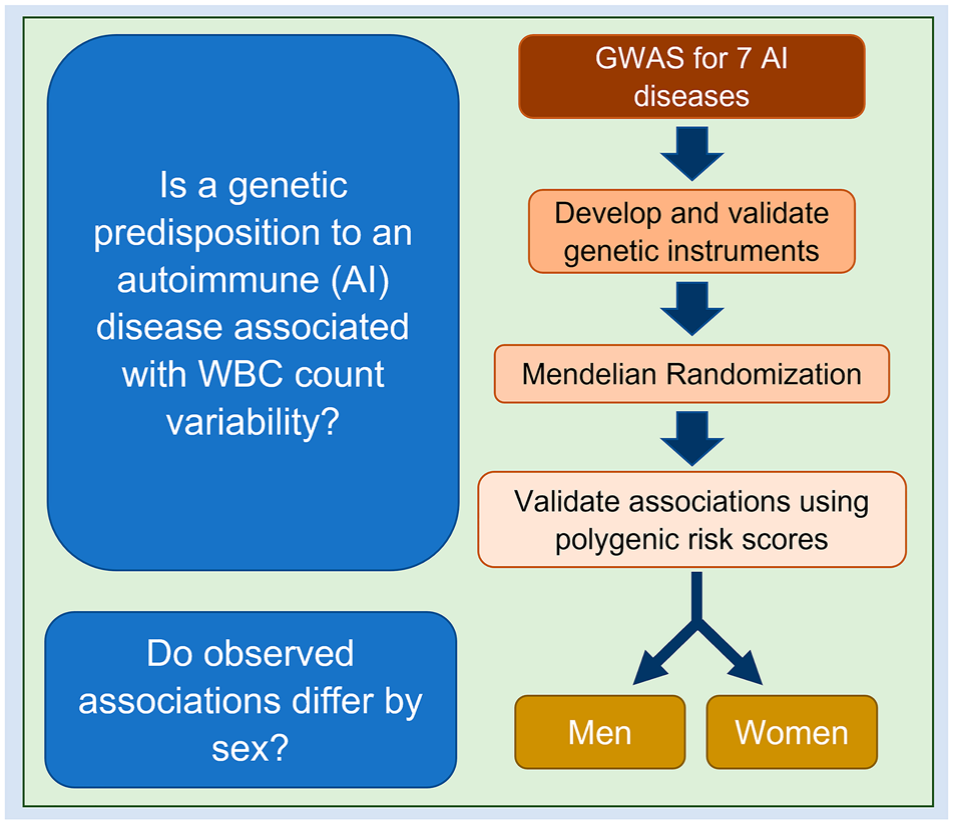
Summary of the research question and study design. Summary level data from genome-wide association studies (GWAS) were extracted for 7 autoimmune (AI) diseases to develop and validate genetic instruments by testing their association with the respective AI disease in BioVU using disease-specific polygenic risk scores (PRS) and PheCodes. Validated instruments were used as exposures in the Mendelian randomization (MR) analyses to test the genetic association between AI diseases and white blood cells (WBCs). Significant association in the MR analyses were validated by testing the association between disease specific PRS and transformed measured WBC counts in ARIC and BioVU. Stratified analyses by sex were performed to measure the magnitude of association in males and females.

Figure 1 summarizes the research questions and study design.

### Study population

Individual-level phenotype data were obtained from BioVU, the Vanderbilt University Medical Center’s (VUMC) Biobank. A detailed description of BioVU has been published elsewhere^21^. Briefly, BioVU accrues DNA from samples obtained during routine clinical care from patients who have consented to have a DNA sample stored. DNA is extracted from discarded samples and linked to a de-identified version of the electronic health record (EHR) at VUMC. Individuals included in this retrospective observational study were previously genotyped on the Illumina Infinium Multi Ethnic Genotyping Array (MEGA^EX^) platform (described below) as part of a broad-based institutional genotyping initiative. The analyses were restricted to participants between 18 and 65 years of age of White European Ancestry, as determined by HAPMAP reference populations and principal components. All participants included in the WBC count analyses had at least one WBC count recorded in their EHR collected on the same day as a health maintenance exam (a wellness visit for routine clinical care).

The Atherosclerosis Risk in Communities (ARIC) study comprised 8926 unrelated genotyped adults of White European Ancestry aged between 45 and 64 years old and followed from 1986 through 2015^22^. ARIC data were obtained from dbGaP (phs000280).

Approval for the present study was obtained from the VUMC Institutional Review Board.

The BioVU and ARIC cohorts represent a clinical- and community-based cohort, respectively. It would be expected that AI diseases would be enriched in a clinical cohort, as compared to a community cohort in which the prevalence of the diseases would be expected to be more comparable to the general population.

### Phenotype data

In BioVU, WBC counts were extracted from the EHR. Analyses were restricted to WBC counts between 1.5 and 25 × 1000 cells/mm^3^ collected as part of a “health maintenance” exam (defined as a clinical encounter associated with a wellness visit denoted by any of the following: ICD-9/ICD-10 codes V70.9, V20, V20.1, V20.2, V70, V70.0, Z00.8, Z00.129, Z00.00). Age was defined as the age at the time of WBC measurements. For participants with multiple WBC measurements (83.8%), the median value was used. Age was the median age of those measurements. Clinical diagnoses for the AI diseases were defined using PheCode phenotypes (https://phewas.mc.vanderbilt.edu/) which are collections of related ICD-9/10-CM (International Classification of Disease) diagnosis codes^23,24^. Cases were defined as individuals with two or more instances of a PheCode in their medical record^25,26^. Controls were defined as those without the PheCodes. For RA, we also excluded those with Juvenile RA among the controls. The following PheCodes were employed—SLE (695.40, 695.41, 695.42), RA (714.1), UC (555.20, 555.21), CD (555.1), MS (335), T1D (250.10, 250.11, 250.12, 250.13, 250.14, 250.15), AIT (245.21).

In the ARIC cohort, WBC counts collected at visit 1 and whose value fell within the range used for BioVU, between 1.5 and 25 × 1000 cells/mm^3^, were examined.

### Genetic data

SNP genotyping of BioVU subjects was performed in the Illumina Infinium Multi-Ethnic Genotyping Array (MEGA^EX^) platform. Quality control (QC) analyses used the PLINK v1.90β3 software^27^. One of each pair of individuals with a second or higher degree of relatedness were excluded. Prior to imputation, genetic data were filtered and standardized through the HRC-1000G-check tool v4.2.5 (http://www.well.ox.ac.uk/~wrayner/tools/) and pre-phased using Eagle v2.4.1^28^. Principal components (PCs) were calculated using the SNPRelate package^29^. Data were imputed using the Michigan Imputation Server in conjunction with the 10/2014 release of the 1000 Genomes cosmopolitan reference haplotypes^30^. Imputed data were filtered for a sample missingness rate < 2%, a SNP missingness rate < 4% and SNP deviation from Hardy–Weinberg P-value < 10^–6^. After QC, 7,585,258 SNPs were available for analysis. Polygenic risk scores were calculated using PLINK v2^31^. Quality control for the ARIC data set was according to the guidelines in the dbGaP release and used PLINK version 1.07, to obtain a sample of unrelated individuals^27^. ARIC data underwent the same QC and imputation protocol as BioVU.

### GWAS summary statistics

Summary statistics for SLE (7219 cases and 15,991 controls)^3^, RA (29,880 cases and 73,758 controls)^4^, UC (4176 cases and 9500 controls)^5^, CD (4474 cases and 9500 controls)^5^, MS (9772 cases and 17,376 controls)^32^, T1D (18,942 cases and 50,1638 controls)^33^, and AIT (15,654 cases and 379,986 controls)^34^ were obtained from publicly available large scale GWAS performed on individuals of European ancestry and were used to define genetic instruments to construct polygenic risk scores (PRS) for each AI disease. The WBC GWAS summary statistics were from a European ancestry subset of a study of blood cell traits in 746,667 participants across 5 global populations and was used the test the associations with the a forementioned seven AI diseases^35^”.

### Analysis

For each AI disease, genetic instruments were constructed using SNP associations derived from GWAS studies. Independent SNPs were selected using a pruning-and-thresholding algorithm that selected an LD-reduced set of SNPs (r^2^ < 0.05)^36^ with a minor allele frequency (MAF) > 5% and an association P-value < 5 × 10^–8^ using Plink V2.

A Mendelian randomization (MR) framework was used to test for associations between genetic instruments for AI diseases and WBC count. MR is an instrumental variable approach used to explore etiological relationships between exposures and outcomes^37–39^. It employs SNPs associated with a chosen exposure as instrumental variables that define the direction and magnitude of associations between the exposure/risk factor and a chosen outcome^38,39^. The general assumptions of the MR approach requires that the genetic instruments (1) are associated with the exposure or risk factor of interest, (2) are not associated with confounders of the exposure-outcome association, and (3) are not associated with the outcome conditional on the risk factor and confounders^38,39^.

The first assumption that the genetic instrument was associated with the AI disease of interest was ascertained by testing its association with cases and controls for the corresponding clinical diagnosis in BioVU using PheCode phenotypes^24^. A polygenic risk score (PRS) was computed for each AI disease by summing the product of each SNP effect size and the SNP dosage in BioVU participants. The PRS was standardized to have a mean of 0 and a standard deviation (SD) of 1. The association between the PRS and the PheCode phenotype was tested using a logistic regression model adjusted for sex, median age, and 10 PCs as covariates. Associations represent odds-ratios per 1 SD change in the PRS. An association P-value < 0.05 was considered significant.

A two-sample MR approach was used to test the association between selected AI diseases and WBC count using inverse-variance weighted regression (IVWR). To ensure that significant associations were not due to pleiotropy, sensitivity analyses were performed using the pleiotropy-robust MR-Egger and weighted median methods to confirm the magnitude and direction of associations^38–40^. When horizontal pleiotropy was detected, MR-PRESSO was applied to assess and correct for horizontal pleiotropy through outlier removal^41^. Association analyses were performed using the genetic instrument for each AI disease as the exposure, and SNPs from the GWAS of WBC count as the outcome. Effect sizes represent the change in transformed WBC count per change in the log odds-ratio of the AI disease. A Bonferroni-adjusted association P-value ≤ 0.05/7 (0.007) for the IVWR effect size was considered significant. In secondary analyses, we examined genetic instruments that were specific to the AI disease of interest by excluding shared SNPs with any of the other AI diseases at P-value < 5 × 10^–4^. All MR analyses were performed using the Mendelian Randomization R package^37^.

Significant associations from the MR analyses were validated by testing the associations between the PRS for the associated AI disease and measured WBC counts in two independent data sets (ARIC and BioVU). Because the distribution of WBC counts was skewed, log transformed WBC counts were the primary outcome variable. The PRS was computed, as described above. The association between log transformed WBC counts and the PRS was tested using a linear regression model adjusted for sex, age, and 10 PCs as covariates. A Bonferroni-adjusted P-value (< 0.05/# AI diseases tested) was considered significant. Sex-stratified analyses were also performed.

The BioVU population included prevalent cases of the selected AI diseases. Thus, our primary analysis was performed among participants without a recorded diagnosis of the of the AI diseases of interest. Cases definitions were based on PheCode phenotypes^24,25^, as described above.

Data are shown as frequency (percentage) for categorical variables and median [interquartile range] for continuous variables; and associations are shown as effect size (95% confidence interval).

### Ethics approval and consent to participate

Per Vanderbilt University IRB approval.

## Results

### Study design

Figure 1 summarizes the study design. GWAS summary data were extracted to develop genetic instruments for the seven AI diseases. We constructed polygenic risk scores for each AI disease and validated them by testing their association with their respective disease in BioVU. Cases were defined by two or more PheCodes for the AI disease. Validated genetic instruments were used as exposures in the MR analyses to test the genetic association between each AI disease and WBC counts using summary GWAS level data. If the AI disease was significantly associated with WBC counts in the MR analyses, the relationship between the PRS for the AI disease and measured WBCs counts was studied in ARIC and BioVU; and stratified analyses by sex were performed.

### Validation of the genetic instruments for each AI disease

To confirm that the selected SNPs were associated with the respective exposure (and thus valid instruments for use in the MR), we constructed SNP-based genetic instruments for each AI disease and tested their associations with cases and controls for the respective disease. Each PRS was significantly associated with the corresponding AI diagnosis in BioVU (P-value < 1 × 10^–10^ in all 7 AI diseases, Supplementary Table 1).

### Mendelian randomization

**Figure 2:**
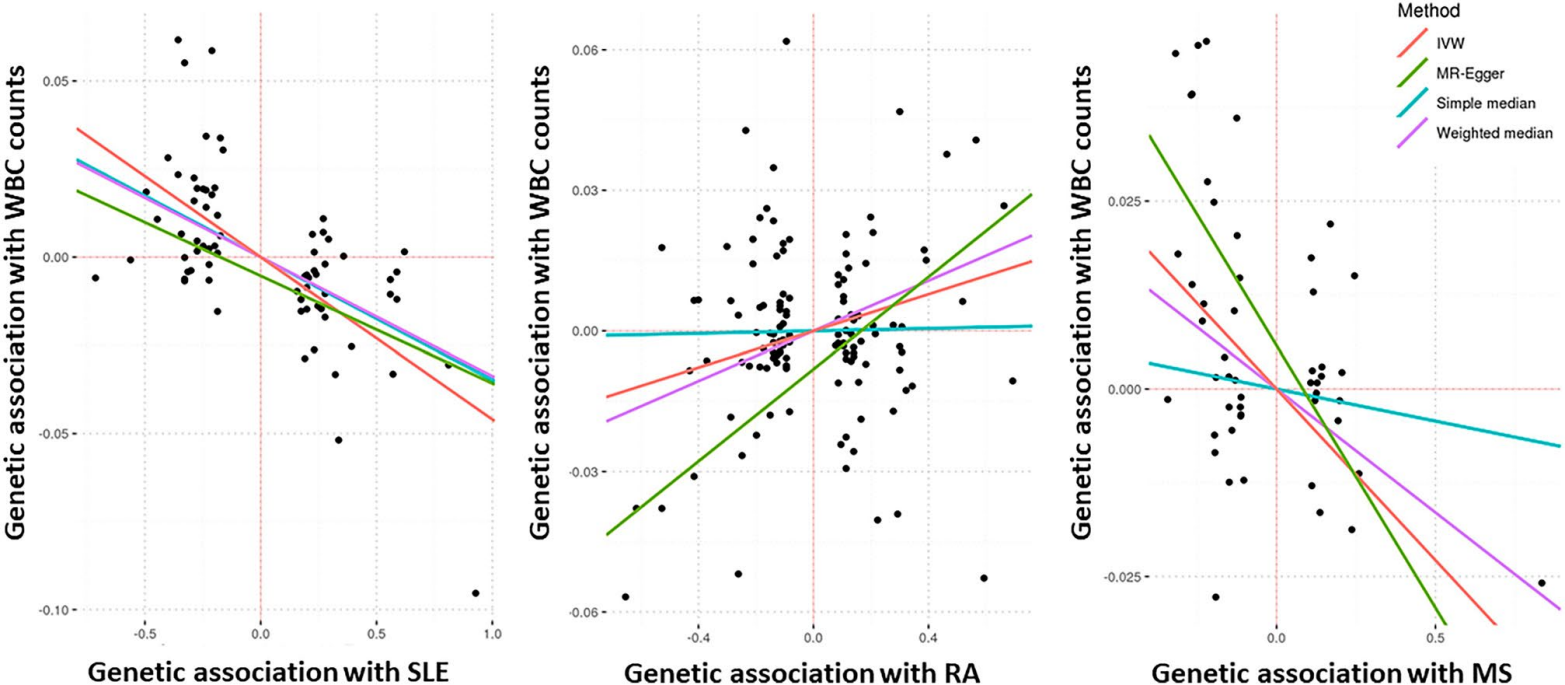
Scatter plots of the inverse-variance weighted regression for systemic lupus erythematosus (SLE), rheumatoid arthritis (RA), and multiple sclerosis (MS) with WBC counts (log transformed). X axes represent the log-odds of the genetic instruments on disease risk (SLE, RA, and MS); and Y axes represent the effect of the genetic instruments in transformed white blood cell (WBC) counts. *IVW* inverse-variance weighted regression.

In the IVWR analysis, three genetic instruments were significantly associated (P-value < 0.007) with WBC counts: SLE, MS, and RA (Fig. 2, Table 1). For each 1 unit increase in the log-odds of a diagnosis of SLE, there was a corresponding decrease of − 0.05 (95% CI − 0.06, − 0.03) units change in transformed WBC count. The change for MS was − 0.05 (95% CI − 0.07, − 0.02) and for RA was 0.02 (95% CI 0.01, 0.03) (Table 1). Sensitivity analyses for RA identified a significant association with the MR-Egger intercept (P-value = 0.001), suggesting a possible bias in the IVWR estimate. The MR-Egger association statistic for RA was 0.05 (95% CI 0.03 0.07) (Supplementary Table 2). The MR-PRESSO analysis for RA identified 47 outliers that inflated the estimated by 91% (P < 2 × 10^–6^). The exclusion of these outliers returned an association statistic of 0.010 (95% CI 0.003, 0.02), P = 0.005.

**Table 1:** Genetic associations between genetic instruments for 7 common autoimmune diseases and white blood cell counts using inverse variance weighted (IVWR) method.

Exposure	#SNPs^1^	Estimate^2^	95% CI	P-value	Het P-value^3^
Systemic lupus erythematosus	71	− 0.05	(− 0.06, − 0.03)	4.5 × 10^–12^	0
Multiple sclerosis	50	− 0.05	(− 0.07, − 0.02)	1 × 10^–4^	0
Rheumatoid arthritis	128	0.02	(0.01, 0.03)	0.006	0
Crohn’s disease	129	0.02	(− 0.0002, 0.04)	0.05	0
Autoimmune thyroiditis	24	− 0.04	(− 0.08, 0.01)	0.15	0
Type 1 diabetes	185	− 0.01	(− 0.02, 0.003)	0.19	3 × 10^–5^
Ulcerative colitis	91	− 0.01	(− 0.03, 0.01)	0.40	0

*CI* confidence interval.

^1^Number of single nucleotide polymorphisms (SNPs) included in the genetic instrument.

^2^Change in transformed white blood cell count per unit increase in the log-odds of the autoimmune disease.

^3^Cochran’s P-value.

### Polygenic risk score (PRS)

We tested associations between a PRS for SLE, MS, and RA and measured WBC counts in a community-based cohort (ARIC). The ARIC population included 8,926 individuals, 47% were male with a median age of 54 [IQR: 49, 59] years, and a median WBC count of 6.0 × 10^3^ [5.0, 7.3] cells/mm^3^. A higher PRS for SLE and MS was associated with lower log-transformed WBC count (SLE: − 0.01, 95% CI − 0.017, − 0.005 per SD increase in the PRS; MS: − 0.01, 95% CI − 0.017, − 0.005) (Table 2). While the direction of association was consistent with the results in the IVWR analyses, it was not significant for RA (0.004, 95% CI − 0.001, 0.010).

**Table 2:** Associations between autoimmune polygenic risk scores and measured transformed white blood cell counts in ARIC and BioVU.

Cohort	Genetic instrument	Estimate^1^	(95% CI)	*P*-value
ARIC	Systemic lupus erythematosus	− 0.010	(− 0.017, − 0.005)	0.0002
	Multiple sclerosis	− 0.010	(− 0.017, − 0.005)	0.0002
	Rheumatoid arthritis	0.004	(− 0.001, 0.010)	0.14
BioVU^2^	Systemic lupus erythematosus	− 0.010	(− 0.013, − 0.007)	3.8 × 10^–14^
	Multiple sclerosis	− 0.008	(− 0.010, − 0.005)	1.2 × 10^–6^
	Rheumatoid arthritis	0.003	(− 0.0001, 0.006)	0.05

*CI* confidence interval.

^1^Change in log transformed white blood cell count per standard deviation change in the polygenic risk score odds of the autoimmune disease, adjusted by sex, age, and 10 principal components.

^2^BioVU included only participants without a diagnosis of each autoimmune disease.

We performed similar analyses in a medical center-derived cohort (BioVU). The BioVU population included 41,442 individuals, 40% were male with a median [IQR] age of 49 [36, 57] years, and a median [IQR] WBC count of 7.5 × 10^3^ [6.1, 9.3] cells/mm^3^ (Supplementary Table 3). Among those with > 1 WBC count measurement, the median [IQR] duration between first and last measurements was 7.0 [2.4–12.3]. After excluding cases for each respective AI disease, we found similar associations with PRS for the 3 AI diseases in BioVU compared to the ARIC cohort (Table 2), with modest improvement of the estimates when cases and non-cases were analyzed together (Supplementary Table 4).

**Figure 3:**
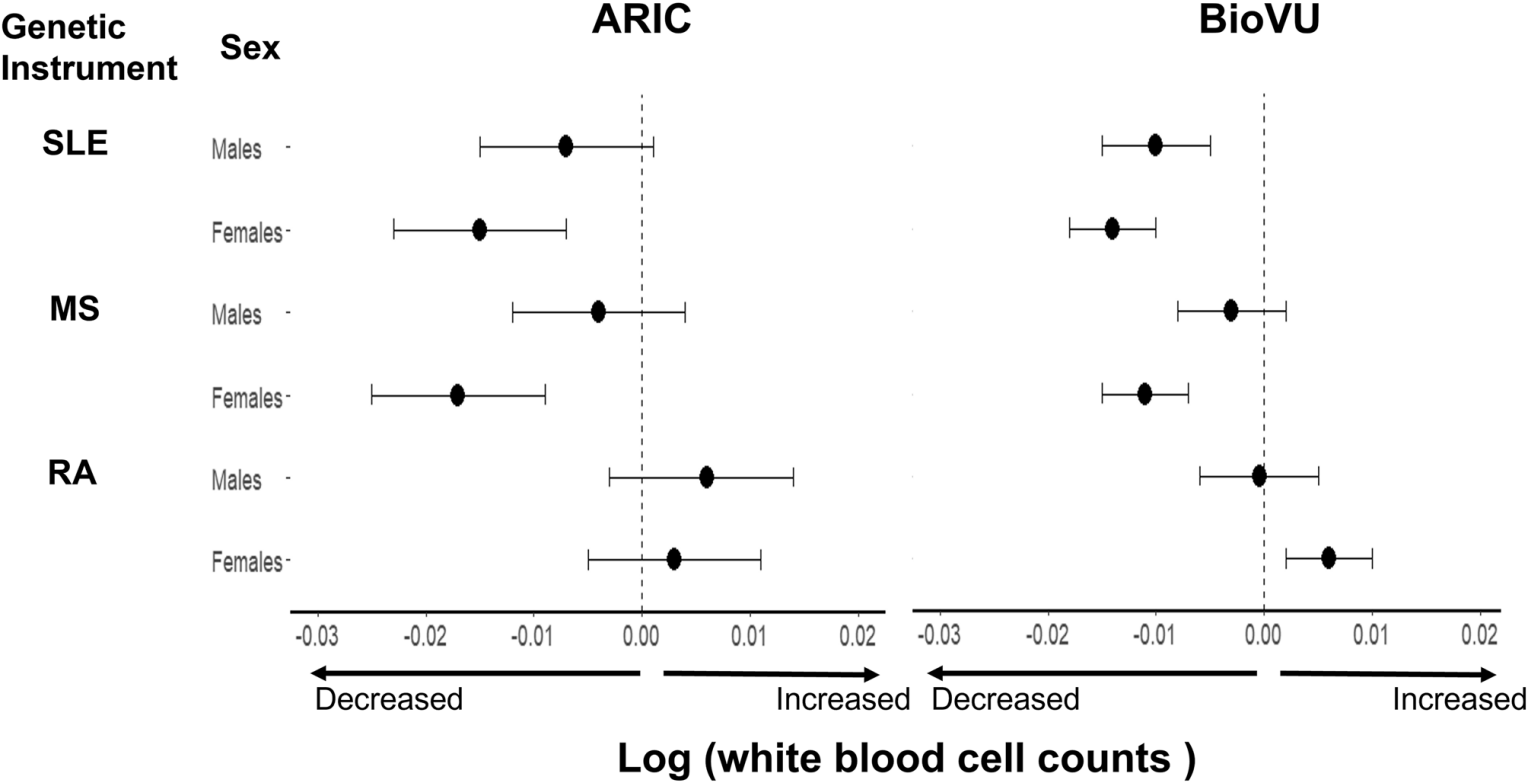
Association between polygenic risk scores for select autoimmune diseases and log transformed white blood cell counts, by sex, in ARIC and BioVU. Participants with a diagnosis of the respective autoimmune disease were excluded in BioVU. *SLE* systemic lupus erythematosus, *MS* multiple sclerosis, *RA* rheumatoid arthritis.

To define if the PRS for the 3 AI diseases exhibit the same association with WBCs in males and females, we first confirmed the association of the PRS with their respective disease in both sexes (Supplementary Table 5). Then, we examined the magnitude of the point estimates for associations between the PRS for the 3 AI diseases and measured WBC counts stratified by sex. In the ARIC cohort, the effect size per SD change in the PRS was larger for females for SLE and MS, but not for RA (Fig. 3, Supplementary Table 6). A similar pattern was seen in BioVU, except that the effect sizes were larger for females for each AI disease (Fig. 3).

## Discussion

We studied the association between polygenic predictors of select AI diseases and WBC counts. Associations examined using a MR framework (IVWR analysis) showed that polygenic predictors for SLE and MS were inversely associated with WBC counts, while a polygenic predictor for RA showed a positive association with WBC counts. Consistent genetic associations were also observed using directly measured WBC counts in a cohort where the prevalence of AI diseases would be expected to be very low (ARIC) and in a clinical population where individuals with known AI diseases were excluded (BioVU). These associations were primarily seen among women, in whom incidence rates of these diseases are known to be higher.

In support of the validity of these findings, we showed the genetic instruments were associated with the expected phenotype; in addition, we tested for horizontal pleiotropy and calculated a new estimated excluding outliers in our sensitivity analysis. We took a two-step approach to understand the relationship between AI diseases and WBC count. First, we used MR as a screen to determine whether a change in WBC count is a down-stream consequence of an AI diseases. Then, we extended these findings by examining the associations between a polygenic risk score and measured WBC counts in subpopulations derived from a hospital-based (BioVU) a community-based (ARIC) cohort.

AI diseases associate with higher or lower levels of WBCs through differing mechanisms. Reduced WBC counts are observed in up to 50% of individuals with SLE and is one symptom contributing to the classification criteria^17,42,43^. The WBC subtypes most impacted are neutrophils and lymphocytes^13,17^, and both lymphopenia (low lymphocyte counts) and neutropenia (low neutrophil counts) can be caused by several factors including disease severity^13,17^ as well as autoantibodies directed against these cells^44–46^. In addition, lower WBC counts are secondarily associated with the use of immunomodulating treatments, hypersplenism and viral infections^46,47^.

While MS is typically not associated with WBC abnormalities, an increase in WBC counts at disease onset and relapse was observed in individuals with MS compared to matched healthy controls in a small clinical study, but this increase did not correlate with disease activity^48^.

RA, on the other hand, is associated with mildly elevated WBC counts, and levels often correlate with active disease^14,49^. The higher WBC counts in RA are likely due to elevated levels of neutrophils, which are important mediators of inflammation in the joint, a characteristic of this disease^14^. WBC count increases may also be secondary to treatment with corticosteroids^50^. A rare complication of RA is Felty syndrome, which is associated with neutropenia and splenomegaly^51,52^.

For RA and SLE, the directions of the genetic associations observed in this study are consistent with clinical observations. In general, the association between SLE genetics and WBC counts was the most robust across populations, which is consistent with the observation that low WBC counts are a common feature of this disease. The inverse association between MS and WBC counts was also consistently observed across data sets, which was not anticipated, as this has not been observed to be a robust pattern of association in epidemiological studies. There are several plausible explanations for the observed associations between the genetic predisposition to some AI diseases and WBC counts.

First, it can be argued that our study population is enriched by cases of these AI diseases. SLE is an uncommon disease that occurs more frequently in women compared to men. The prevalence rates among individuals of white race are ~ 8.5/10,000 for women and ~ 0.9/10,000 for men^16^. Thus, in a community cohort such as the ARIC data set, it would not be expected to find an association between a genetic predisposition for SLE and a clinical symptom found in some SLE patients given that in general, PRS are relatively weak proxies of disease risk and that fewer than 5 cases of SLE would be expected in this small cohort of ~ 9000 individuals. Moreover, RA is a relatively more common disease with sex-specific prevalence rates of ~ 2.6/1000 men and ~ 7.1/1000 women^18^ but no significant association was found for genetic susceptibility for RA in ARIC, suggesting that case enrichment is an unlikely explanation.

Another explanation is that the polygenic predictors of these AI diseases capture genetic mechanisms that modulate WBC levels that are constitutively active. This could be plausible for SLE, where a low WBC is one of the diagnostic criteria for disease^43^. Individuals with a genetic predisposition toward lower WBCs are more likely to satisfy the diagnostic criteria. Consequently, a GWAS of SLE and a PRS derived from this GWAS could contain SNPs that associate with WBC, independent of SLE disease status. However, for MS and RA, changes in WBCs are not a stereotypical finding, making this a less likely explanation for the associations observed with these diseases.

Genetic disease risk represents a continuous risk spectrum. Under this scenario, all clinical features (including WBC effects) are manifest among individuals in proportion to the dose of risk alleles they carry. This is true for the genetics underlying many continuous traits, such as height^53^. With AI diseases, it is thought that an environmental trigger is required to produce disease-related autoantibodies, immune dysregulation, and subsequent disease expression^7,54^. Thus, in the absence of a trigger (and thus absence of disease), an association between a genetic predisposition and disease-associated biomarkers would not be expected.

It is also possible that the prevalence of subclinical or incomplete AI disease may be higher than population estimates would suggest, and a polygenic predictor is effective at detecting subclinical disease associations when examining a continuous disease feature. Under this scenario, it would be expected that effect sizes (such as the magnitude of the association with WBC levels), would be greater among women, who have higher incidence rates, as compared to men. Consistently, effects sizes were larger for women for each of the disease examined. A gender differential would not be expected if the PRS were capturing constitutive regulation of WBC levels, as this activity should be comparable across genders. Based on the findings presented here, we hypothesize that the observed associations between the genetic predictors for these 3 AI diseases and WBC counts across these populations is explained by some degree of immune dysfunction and/or subclinical disease that is translated in modest changes in WBC counts. However, we cannot rule out that the PRS are far more sensitive than we would otherwise assume, and it is able to detect associations in populations with very low numbers of cases.

This study has limitations. While we excluded individuals in the BioVU population with known AI diseases, it is possible that these diseases were represented at higher rates than expected in the study populations. In addition, if individuals with a disease were under active treatment with medications that reduced or increased WBC levels, this could lead to larger or smaller (depending on drug and disease) effects sizes in the PRS associations that may be detectable among smaller numbers of cases. Finally, these analyses were restricted to individuals of White European Ancestry. For AI diseases like SLE, incidence rates are higher among individuals of African ancestries^55^. Thus, these results are likely not generalizable to other ancestries.

In conclusion, we found that a genetic predisposition toward some AI diseases (SLE, RA and MS) was associated with either lower or higher WBC counts in multiple populations. The directions of associations for SLE and RA were consistent with clinical observations of lower and higher WBC counts, respectively, as were differences in the magnitudes of the associations across sexes.

## Supplementary Information

Supplementary Tables.

## Data Availability

GWAS summary statistics for the 7 autoimmune disease and WBC counts are publicly available in the GWAS catalog. The list of SNPs that were included in the genetic instruments for RA, SLE, and MS are available in Supplementary Table 7. Individual level data from ARIC can be obtained through dbGaP (phs000280), and BioVU data by direct request to the VUMC investigators, pending BioVU approval and data use agreements.

## References

[1] Wang X, Qiu L, Li Z, Wang XY, Yi H (2018). Understanding the multifaceted role of neutrophils in cancer and autoimmune diseases. Front. Immunol..

[2] Herrero-Cervera A, Soehnlein O, Kenne E (2022). Neutrophils in chronic inflammatory diseases. Cell Mol. Immunol..

[3] Bentham J (2015). Genetic association analyses implicate aberrant regulation of innate and adaptive immunity genes in the pathogenesis of systemic lupus erythematosus. Nat. Genet..

[4] Okada Y (2014). Genetics of rheumatoid arthritis contributes to biology and drug discovery. Nature.

[5] de Lange KM (2017). Genome-wide association study implicates immune activation of multiple integrin genes in inflammatory bowel disease. Nat. Genet..

[6] Willame C (2021). Incidence rates of autoimmune diseases in European Healthcare databases: A contribution of the ADVANCE project. Drug Saf..

[7] Barbhaiya M, Costenbader KH (2016). Environmental exposures and the development of systemic lupus erythematosus. Curr. Opin. Rheumatol..

[8] Lambers WM, Westra J, Jonkman MF, Bootsma H, de Leeuw K (2020). Incomplete systemic lupus erythematosus: What remains after application of American College of rheumatology and systemic lupus international collaborating clinics criteria?. Arthritis Care Res. (Hoboken).

[9] Feldman DE (2007). Delay in consultation with specialists for persons with suspected new-onset rheumatoid arthritis: A population-based study. Arthritis Rheum..

[10] Kingwell E (2010). Factors associated with delay to medical recognition in two Canadian multiple sclerosis cohorts. J. Neurol. Sci..

[11] Proceedings of the Diagnostic Criteria in Autoimmune Diseases, 9th International Congress on Autoimmunity, March 26–30, 2014, Nice, France. *J. Autoimmun.* **48–49**, 1–152 (2014). 25275157

[12] Duarte-Garcia A (2022). Rising incidence and prevalence of systemic lupus erythematosus: A population-based study over four decades. Ann. Rheum. Dis..

[13] Kandane-Rathnayake R (2021). Independent associations of lymphopenia and neutropenia in patients with systemic lupus erythematosus: A longitudinal, multinational study. Rheumatology (Oxford).

[14] Wright HL, Moots RJ, Edwards SW (2014). The multifactorial role of neutrophils in rheumatoid arthritis. Nat. Rev. Rheumatol..

[15] Hayter SM, Cook MC (2012). Updated assessment of the prevalence, spectrum and case definition of autoimmune disease. Autoimmun. Rev..

[16] Izmirly PM (2021). Prevalence of systemic lupus erythematosus in the United States: Estimates from a meta-analysis of the centers for disease control and prevention national lupus registries. Arthritis Rheumatol..

[17] Dubois EL, Tuffanelli DL (1964). Clinical manifestations of systemic lupus erythematosus. Computer analysis of 520 cases. JAMA.

[18] Hunter TM (2017). Prevalence of rheumatoid arthritis in the United States adult population in healthcare claims databases, 2004–2014. Rheumatol. Int..

[19] Cotsapas C (2011). Pervasive sharing of genetic effects in autoimmune disease. PLoS Genet..

[20] Ramos PS (2011). A comprehensive analysis of shared loci between systemic lupus erythematosus (SLE) and sixteen autoimmune diseases reveals limited genetic overlap. PLoS Genet..

[21] Roden DM (2008). Development of a large-scale de-identified DNA biobank to enable personalized medicine. Clin. Pharmacol. Ther..

[22] The ARIC Investigators (1989). The atherosclerosis risk in communities (ARIC) study: Design and objectives. Am. J. Epidemiol..

[23] Wei WQ (2016). Combining billing codes, clinical notes, and medications from electronic health records provides superior phenotyping performance. J. Am. Med. Inf. Assoc..

[24] Wu P (2019). Mapping ICD-10 and ICD-10-CM codes to phecodes: Workflow development and initial evaluation. JMIR Med. Inform.

[25] Wei WQ (2017). Evaluating phecodes, clinical classification software, and ICD-9-CM codes for phenome-wide association studies in the electronic health record. PLoS ONE.

[26] Denny JC (2013). Systematic comparison of phenome-wide association study of electronic medical record data and genome-wide association study data. Nat. Biotechnol..

[27] Purcell S (2007). PLINK: A tool set for whole-genome association and population-based linkage analyses. Am. J. Hum. Genet..

[28] Loh PR (2016). Reference-based phasing using the Haplotype Reference Consortium panel. Nat. Genet..

[29] Zheng X (2012). A high-performance computing toolset for relatedness and principal component analysis of SNP data. Bioinformatics.

[30] Das S (2016). Next-generation genotype imputation service and methods. Nat. Genet..

[31] Chang CC (2015). Second-generation PLINK: Rising to the challenge of larger and richer datasets. Gigascience.

[32] International Multiple Sclerosis Genetics Consortium (2011). Genetic risk and a primary role for cell-mediated immune mechanisms in multiple sclerosis. Nature.

[33] Chiou J (2021). Interpreting type 1 diabetes risk with genetics and single-cell epigenomics. Nature.

[34] Sakaue S (2021). A cross-population atlas of genetic associations for 220 human phenotypes. Nat. Genet..

[35] Chen MH (2020). Trans-ethnic and ancestry-specific blood-cell genetics in 746,667 individuals from 5 global populations. Cell.

[36] Zhu Z (2018). Causal associations between risk factors and common diseases inferred from GWAS summary data. Nat. Commun..

[37] Yavorska OO, Burgess S (2017). MendelianRandomization: An R package for performing Mendelian randomization analyses using summarized data. Int. J. Epidemiol..

[38] Burgess S (2019). Guidelines for performing Mendelian randomization investigations. Wellcome Open Res..

[39] Burgess S, Thompson SG (2017). Interpreting findings from Mendelian randomization using the MR-Egger method. Eur. J. Epidemiol..

[40] Bowden J, Davey Smith G, Haycock PC, Burgess S (2016). Consistent estimation in Mendelian randomization with some invalid instruments using a weighted median estimator. Genet. Epidemiol..

[41] Verbanck M, Chen CY, Neale B, Do R (2018). Detection of widespread horizontal pleiotropy in causal relationships inferred from Mendelian randomization between complex traits and diseases. Nat. Genet..

[42] Aringer M (2019). 2019 European League against Rheumatism/American College of Rheumatology Classification criteria for systemic lupus erythematosus. Arthritis Rheumatol..

[43] Tan EM (1982). The 1982 revised criteria for the classification of systemic lupus erythematosus. Arthritis Rheum..

[44] Hoffman IE (2004). Specific antinuclear antibodies are associated with clinical features in systemic lupus erythematosus. Ann. Rheum. Dis..

[45] Fredi M (2014). Rare autoantibodies to cellular antigens in systemic lupus erythematosus. Lupus.

[46] Martin M, Guffroy A, Argemi X, Martin T (2017). Systemic lupus erythematosus and lymphopenia: Clinical and pathophysiological features. Rev. Med. Intern..

[47] Fayyaz A (2015). Haematological manifestations of lupus. Lupus Sci. Med..

[48] Akaishi T (2021). White blood cell count profiles in multiple sclerosis during attacks before the initiation of acute and chronic treatments. Sci. Rep..

[49] Wipke BT, Allen PM (2001). Essential role of neutrophils in the initiation and progression of a murine model of rheumatoid arthritis. J. Immunol..

[50] Fauci AS, Dale DC, Balow JE (1976). Glucocorticosteroid therapy: Mechanisms of action and clinical considerations. Ann. Intern. Med..

[51] Bradley JD, Pinals RS (1983). Felty’s syndrome presenting without arthritis. Clin. Exp. Rheumatol..

[52] Burks EJ, Loughran TP (2006). Pathogenesis of neutropenia in large granular lymphocyte leukemia and Felty syndrome. Blood Rev..

[53] Yengo L (2018). Meta-analysis of genome-wide association studies for height and body mass index in approximately 700000 individuals of European ancestry. Hum. Mol. Genet..

[54] Selmi C, Lu Q, Humble MC (2012). Heritability versus the role of the environment in autoimmunity. J. Autoimmun..

[55] Lewis MJ, Jawad AS (2017). The effect of ethnicity and genetic ancestry on the epidemiology, clinical features and outcome of systemic lupus erythematosus. Rheumatology (Oxford).

