# Supplementary material for: Genetic susceptibility for autoimmune diseases and white blood cell count
Source: Sci Rep. 2023 Apr 11;13:5852. doi: 10.1038/s41598-023-32799-8 (PMC10090175; doi:10.1038/s41598-023-32799-8)
Supplement: Supplementary file 1 — Supplementary Tables. [file 41598_2023_32799_MOESM1_ESM.docx]

**Supplementary material:**

1. **Supplementary Table 1:** Association between genetic instruments for 7 autoimmune diseases and their respective disease diagnosis in BioVU.
2. **Supplementary Table 2:** Egger and weighted median mendelian randomization results for genetic instruments for 7 autoimmune diseases and log transformed white blood cell counts
3. **Supplementary Table 3**: Characteristics by autoimmune disease status in the BioVU cohort.
4. **Supplementary Table 4:** Association for polygenic risk scores (PRS) with white blood cell counts among individuals with (Cases) and without (non-cases) disease in the BioVU cohort.
5. **Supplementary Table 5**: Supplementary Table 5: Sex-stratified analyses of the association for autoimmune polygenic risk scores with their respective disease diagnosis in BioVU
6. **Supplementary Table 6**: Sex-stratified analyses of the association for autoimmune polygenic risk scores with log transformed white blood cell counts in the ARIC cohort and in non-cases in the BioVU cohort.
7. **Supplementary Table 7**: Genetic instruments for systemic lupus erythematosus (SLE), Rheumatoid arthritis (RA) and multiple sclerosis (MS)

.

| **Supplementary Table 1:** Association between genetic instruments for 7 autoimmune diseases and their respective disease diagnosis in BioVU | | | | |
| --- | --- | --- | --- | --- |
| **Autoimmune disease diagnosis** | **Instrument** | **# Cases** | **OR (95%CI)** | **P-value** |
| Systemic lupus erythematosus (SLE) | SLE | 880 | 1.48 (1.39, 1.57) | 9.2x10^-33^ |
| Multiple sclerosis (MS) | MS | 1192 | 1.58(1.49, 1.68) | 1.2x10^-52^ |
| Rheumatoid arthritis (RA) | RA | 1623 | 1.46 (1.38, 1.55) | 1.9x10^-51^ |
| Crohn’s disease (CD) | CD | 1579 | 1.90(1.79, 2.01) | 2.0x10^-122^ |
| Autoimmune thyroiditis (AIT) | AIT | 592 | 1.48(1.37, 1.60) | 2.6x10^-71^ |
| Type 1 diabetes (T1D) | T1D | 1881 | 1.46(1.41, 1.52) | 3.9x10^-71^ |
| Ulcerative colitis (UC) | UC | 845 | 1.72 (1.59, 1.86) | 1.9x10^-36^ |

A high P-value reflects validity of the genetic instrument to predict the autoimmune disease of interest. OR (95%CI): Odds ratio (95% confidence interval)

| **Supplementary Table 2.** Egger and weighted median mendelian randomization results for genetic instruments for 7 autoimmune diseases and log transformed white blood cell counts | | | | | | | | | |
| --- | --- | --- | --- | --- | --- | --- | --- | --- | --- |
| **AI** | **#SNPs^a^** |  | **Egger** | | |  | **Weighted Median** | | |
|  |  |  | **Estimate (95%CI)** | **P-value** | **P-value^b^** |  | **Estimate (95% CI)** | **P-value** | |
| SLE | 71 |  | -0.03 (-0.06, -0.0002) | 0.05 | 0.26 |  | -0.03 (-0.04, -0.03) | 1.7x10^-22^ |  |
| MS | 50 |  | -0.07 (-0.12, -0.02) | 0.003 | 0.24 |  | -0.03 (-0.04, -0.03) | 2.5x10^-29^ |  |
| RA | 128 |  | 0.05 (0.03, 0.07) | 2x10^-05^ | 0.001 |  | 0.03 (0.02, 0.03) | 2.8x10^-14^ |  |
| CD | 129 |  | -0.01 (-0.05, 0.04) | 0.81 | 0.29 |  | 0.004 (-0.002, 0.01) | 0.17 |  |
| AIT | 24 |  | -0.07 (-0.22, 0.08) | 0.36 | 0.63 |  | -0.03 (-0.05, -0.02) | 8.8x10^-09^ |  |
| T1D | 185 |  | -0.01 (-0.03, 0.01) | 0.18 | 0.47 |  | -0.01 (-0.01, -0.01) | 6.7x10^-06^ |  |
| UC | 91 |  | -0.01 (-0.08, 0.07) | 0.90 | 0.87 |  | 0.003 (-0.004, 0.01) | 0.40 |  |
| Outcome: GWAS summary statistics for log transformed white blood cell counts. Exposure: genetic instruments for an autoimmune disorder (AI): SLE – systemic lupus erythematosus, RA – rheumatoid arthritis, UC – ulcerative colitis, CD – Crohn’s disease, MS – multiple sclerosis, T1D – type 1 diabetes, AIT – autoimmune thyroiditis. CI – confidence interval. ^a^ SNP: number of single nucleotide polymorphisms included the genetic instrument; ^b^ Intercept P-value | | | | | | | | | |

| **Supplementary Table 3:** Characteristics by autoimmune disease status in the BioVU cohort | | | | | | | | | | |
| --- | --- | --- | --- | --- | --- | --- | --- | --- | --- | --- |
|  |  | **Cases** | | | |  | **Non cases** | | | |
| **AI disease** |  | **N** | **Males (%)** | **Age (years)** | **WBC counts (thousand/mm^3^)** |  | **N** | **Males (%)** | **Age (years)** | **WBC counts (thousand/mm^3^)** |
| SLE |  | 981 | 124 (12.6) | 48.2[37.6, 56.4] | 7.0 [5.6, 8.7] |  | 40461 | 16505 (40.8) | 49 [36.2, 57.4] | 7.5 [6.1, 9.3] |
| MS |  | 1385 | 346 (25.0) | 47.8 [39.1, 55.4] | 7.1 [5.8, 8.7] |  | 40009 | 16266 (40.7) | 49.0 [36.1, 57.5] | 7.5 (6.1, 9.3) |
| RA |  | 1479 | 375 (25.4) | 53.7 [45.4, 59.7] | 7.6 [6.3, 9.3] |  | 39876 | 16224 (40.7) | 48.8 [35.9, 57.3] | 7.5 [6.1, 9.3] |
| Data are shown as frequency (percentage) and median [interquartile range] for categorical and continuous variables, respectively. AI: autoimmune; SLE: systemic lupus erythematosus; RA: rheumatoid arthritis; MS: multiple sclerosis; WBC: white blood cells | | | | | | | | | | |

| **Supplementary Table 4** Association for polygenic risk scores (PRS) with white blood cell counts in the BioVU cohort | | | | | | |
| --- | --- | --- | --- | --- | --- | --- |
| **PRS** |  | **All BioVU (cases and non-cases)** | |  | **Cases** | |
|  |  | **Estimate (95%CI)^a^** | **P-value** |  | **Estimate (95%CI)^a^** | **P-value** |
| SLE |  | -0.010 (-0.020, -0.010) | 2.4x10^-17^ |  | -0.030 (-0.050, -0.010) | 3.9x10^-4^ |
| MS |  | -0.09 (-0.012, -0.006) | 3.4x10^-8^ |  | -0.020 (-0.030, -0.001) | 0.04 |
| RA |  | 0.004 (0.001, 0.007) | 0.02 |  | 0.010 (-0.004, 0.030) | 0.17 |
| Cases are defined with the presence of at least 2 or more phecodes for the respective autoimmune disease, and non-cases as those without a phecode for the disease. ^a^ Estimate is defined as change in the log transformed white blood cell counts per 1 standard deviation in the polygenic risk score (PRS); adjusted by sex, median age, and 10 principal components. SLE: systemic lupus erythematosus; RA: rheumatoid arthritis; MS: multiple sclerosis; CI: confidence interval | | | | | | |

| **Supplementary Table 5:** Sex stratified analyses of the association for polygenic risk scores and their respective disease diagnosis in BioVU | | | |
| --- | --- | --- | --- |
| **Autoimmune disease** | **Sex** | **OR (95%CI)** | **P-value** |
| Systemic lupus erythematosus (SLE) | Males | 1.54 (1.29, 1.83) | 1.78x10^-6^ |
|  | Females | 1.46 (1.37, 1.57) | 6.3x10^-28^ |
| Multiple Sclerosis (MS) | Males | 1.65 (1.46, 1.87) | 4.5x10^-16^ |
|  | Females | 1.56 (1.46, 1.67) | 1.9x10^-38^ |
| Rheumatoid Arthritis (RA) | Males | 1.57 (1.4,3 1.72) | 9.3x10^-22^ |
|  | Females | 1.42 (1.34, 1.51) | 3.5x10^-32^ |
| Cases are defined as individuals with the presence of at least 2 or more phecodes for the respective autoimmune disease | | | |

| **Supplementary Table 6**: Sex-stratified analyses of the association for autoimmune polygenic risk scores with log transformed white blood cell counts in the ARIC cohort and in non-cases in the BioVU cohort | | | | | | | | |
| --- | --- | --- | --- | --- | --- | --- | --- | --- |
| **PRS** | **Sex** |  | **ARIC** | |  | **BioVU** | |  |
|  |  |  | **Estimate (95%CI)^a^** | **P-value** |  | **Estimate (95%CI)^a^** | **P-value** |  |
| SLE | Males |  | -0.007 (-0.015, 0.001) | 0.11 |  | -0.010 (-0.015, -0.005) | 0.0001 |  |
|  | Females |  | -0.015 (-0.023, -0.007) | 0.0004 |  | -0.014 (-0.018, -0.010) | 3.6x10^-11^ |  |
| MS | Males |  | -0.004 (-0.012, 0.004) | 0.36 |  | -0.003 (-0.008, 0.002) | 0.23 |  |
|  | Females |  | -0.017 (-0.025, -0.009) | 3.2x10^-5^ |  | -0.011 (-0.015, -0.007) | 6.7x10^-8^ |  |
| RA | Males |  | 0.006 (-0.003, 0.014) | 0.18 |  | -0.0004 (-0.006, 0.005) | 0.890 |  |
|  | Females |  | 0.003 (-0.005, 0.011) | 0.48 |  | 0.006 (0.002, 0.010) | 0.007 |  |
| Non-cases are defined as individuals without a phecode for the disease in BioVU. ^a^estimate is defined as change in the log10 transformed white blood cell counts per 1 standard deviation in the polygenic risk score (PRS); adjusted by sex, median age, and 10 principal components. SLE: systemic lupus erythematosus; RA: rheumatoid arthritis; MS: multiple sclerosis; CI: confidence interval | | | | | | | |  |

| **Supplementary Table 7:** Genetic instruments for systemic lupus erythematosus (SLE), Rheumatoid arthritis (RA) and multiple sclerosis (MS) | | | | | | | | | | | | | | |
| --- | --- | --- | --- | --- | --- | --- | --- | --- | --- | --- | --- | --- | --- | --- |
| **SLE** | Bentham, J. et al. Genetic association analyses implicate aberrant regulation of innate and adaptive immunity genes in the pathogenesis of systemic lupus erythematosus. Nat Genet 47, 1457-1464, doi:10.1038/ng.3434 (2015) | | | | | | | | | | | | | |
| **SNP ID** | **Ch** | **Pos** | **EA** | **OA** | **Beta** | **P-value** |  | **SNP ID** | **Ch** | **Pos** | **EA** | **OA** | **Beta** | **P-value** |
| rs17849501 | 1 | 183542323 | T | C | 0.811 | 1.81E-59 |  | rs555007 | 6 | 31850332 | C | T | -0.494 | 1.38E-15 |
| rs4661543 | 1 | 15229101 | T | G | -0.274 | 9.40E-11 |  | rs670369 | 6 | 138147048 | C | T | 0.223 | 6.83E-9 |
| rs4916215 | 1 | 173314540 | C | T | -0.223 | 5.07E-11 |  | rs28383461 | 6 | 32609607 | A | G | -0.357 | 1.05E-20 |
| rs6671847 | 1 | 161478810 | A | G | 0.199 | 6.64E-12 |  | rs2856816 | 6 | 33045500 | C | T | 0.392 | 9.18E-16 |
| rs6679677 | 1 | 114303808 | A | C | 0.336 | 4.55E-13 |  | rs3129767 | 6 | 32594975 | G | T | 0.571 | 3.00E-71 |
| rs78517564 | 1 | 183442324 | C | A | -0.329 | 1.90E-10 |  | rs389884 | 6 | 31940897 | G | A | 0.928 | 2.93E-102 |
| rs844649 | 1 | 173224343 | C | T | 0.231 | 1.20E-13 |  | rs4713571 | 6 | 32626899 | T | G | -0.400 | 9.73E-32 |
| rs946173 | 1 | 183260659 | G | A | 0.270 | 2.69E-11 |  | rs501480 | 6 | 33563946 | C | T | 0.199 | 2.07E-11 |
| rs10048743 | 2 | 213890232 | G | T | 0.231 | 2.04E-8 |  | rs555007 | 6 | 31850332 | C | T | -0.494 | 1.38E-15 |
| rs10200680 | 2 | 223961877 | T | C | -0.248 | 4.96E-9 |  | rs670369 | 6 | 138147048 | C | T | 0.223 | 6.83E-09 |
| rs1263147 | 2 | 191581941 | G | T | 0.191 | 1.89E-9 |  | rs675640 | 6 | 138127204 | A | C | 0.278 | 1.28E-09 |
| rs13010752 | 2 | 191946603 | A | T | -0.342 | 1.03E-11 |  | rs67575965 | 6 | 26196593 | G | A | 0.231 | 3.60E-12 |
| rs13019891 | 2 | 113829869 | T | G | -0.562 | 1.65E-83 |  | rs6903257 | 6 | 25600191 | T | C | 0.199 | 4.45E-09 |
| rs16833239 | 2 | 191940260 | A | G | -0.446 | 5.81E-14 |  | rs6903535 | 6 | 28417222 | G | A | 0.191 | 1.13E-10 |
| rs2573219 | 2 | 233288667 | C | A | 0.588 | 1.13E-42 |  | rs7451194 | 6 | 32735645 | C | T | -0.288 | 1.71E-19 |
| rs268124 | 2 | 65654364 | C | T | -0.186 | 8.60E-9 |  | rs7454436 | 6 | 32352907 | C | G | 0.322 | 1.60E-10 |
| rs4274624 | 2 | 191958656 | C | T | 0.560 | 9.73E-66 |  | rs7768653 | 6 | 106574794 | C | T | 0.207 | 3.11E-12 |
| rs512681 | 2 | 119450163 | A | C | -0.301 | 8.75E-13 |  | rs805288 | 6 | 31678028 | T | C | -0.288 | 6.77E-18 |
| rs887701 | 2 | 191562927 | A | G | 0.239 | 2.39E-9 |  | rs9258051 | 6 | 29665228 | A | G | -0.163 | 1.88E-08 |
| rs1464446 | 3 | 146601295 | T | G | -0.329 | 2.79E-16 |  | rs9258947 | 6 | 29839524 | C | G | 0.261 | 1.19E-14 |
| rs9852014 | 3 | 129084581 | G | A | 0.621 | 2.26E-36 |  | rs9262619 | 6 | 31022266 | C | G | -0.211 | 1.33E-10 |
| rs13136219 | 4 | 102743687 | T | C | -0.174 | 3.50E-10 |  | rs9263869 | 6 | 31170014 | C | T | -0.198 | 1.48E-10 |
| rs1078324 | 5 | 149202268 | A | C | -0.713 | 7.11E-20 |  | rs9267368 | 6 | 31464321 | A | T | -0.329 | 3.83E-24 |
| rs2431697 | 5 | 159879978 | C | T | -0.223 | 2.60E-14 |  | rs9268830 | 6 | 32427572 | G | C | -0.329 | 2.51E-17 |
| rs28578492 | 5 | 133424804 | G | A | 0.358 | 5.44E-10 |  | rs9368503 | 6 | 27303745 | T | A | 0.174 | 5.88E-09 |
| rs4958436 | 5 | 150442829 | C | T | 0.278 | 2.61E-16 |  | rs9405083 | 6 | 31319489 | T | C | -0.274 | 6.50E-16 |
| 6: 30989906 | 6 | 30989906 | C | T | -0.248 | 6.84E-16 |  | rs9461633 | 6 | 30761168 | G | A | -0.236 | 7.98E-11 |
| rs1055568 | 6 | 31440006 | T | C | -0.174 | 2.34E-8 |  | rs35000415 | 7 | 128585616 | T | C | 0.588 | 1.86E-45 |
| rs13193526 | 6 | 34913186 | A | G | 0.293 | 1.64E-8 |  | rs3807134 | 7 | 128577338 | C | T | -0.274 | 1.72E-08 |
| rs13195522 | 6 | 32656253 | A | C | -0.357 | 2.01E-23 |  | rs6960994 | 7 | 128692657 | C | T | -0.198 | 1.27E-08 |
| rs194675 | 6 | 32905746 | A | T | -0.211 | 2.71E-12 |  | rs2736332 | 8 | 11339965 | C | G | 0.278 | 4.83E-18 |
| rs2022082 | 6 | 30333820 | C | T | -0.236 | 2.78E-12 |  | rs7097397 | 10 | 50025396 | A | G | -0.186 | 8.60E-11 |
| rs2294473 | 6 | 33099166 | G | C | 0.157 | 3.89E-8 |  | rs353608 | 11 | 35101738 | A | G | -0.186 | 2.93E-11 |
| rs2745401 | 6 | 29488478 | G | A | 0.174 | 3.00E-9 |  | rs58688157 | 11 | 625085 | G | A | -0.223 | 2.97E-11 |
| rs28383461 | 6 | 32609607 | A | G | -0.357 | 1.05E-20 |  | rs13332649 | 16 | 85966683 | G | A | -0.315 | 5.43E-17 |
| rs2856816 | 6 | 33045500 | C | T | 0.392 | 9.18E-16 |  | rs35472514 | 16 | 31283323 | G | C | 0.560 | 2.49E-47 |
| rs3129767 | 6 | 32594975 | G | T | 0.571 | 3.00E-71 |  | rs62051483 | 16 | 31327376 | G | A | 0.270 | 4.00E-15 |
| rs389884 | 6 | 31940897 | G | A | 0.928 | 2.93E-102 |  | rs34725611 | 19 | 10477067 | G | A | -0.236 | 1.48E-12 |
| rs4713571 | 6 | 32626899 | T | G | -0.400 | 9.73E-32 |  | rs4820091 | 22 | 21940189 | G | T | 0.247 | 8.30E-14 |
| rs501480 | 6 | 33563946 | C | T | 0.199 | 2.07E-11 |  |  |  |  |  |  |  |  |
| **RA** | Okada, Y. et al. Genetics of rheumatoid arthritis contributes to biology and drug discovery. Nature 506, 376-381, doi:10.1038/nature12873 (2014) | | | | | | | | | | | | | |
| **SNP ID** | **Ch** | **Pos** | **EA** | **OA** | **Beta** | **P-value** |  | **SNP ID** | **Ch** | **Pos** | **EA** | **OA** | **Beta** | **P-value** |
| rs10912602 | 1 | 173341999 | A | G | 0.104 | 4.40E-10 |  | rs115461385 | 6 | 32058676 | T | C | 0.207 | 2.70E-09 |
| rs112429016 | 1 | 113926865 | T | C | 0.157 | 9.60E-13 |  | rs115493740 | 6 | 32838539 | A | G | 0.663 | 6.60E-150 |
| rs12126142 | 1 | 154425456 | A | G | -0.083 | 3.50E-9 |  | rs140465980 | 6 | 29751129 | T | C | -0.174 | 5.30E-23 |
| rs1217401 | 1 | 114438951 | A | G | 0.113 | 5.20E-13 |  | rs144732803 | 6 | 29035159 | G | C | -0.117 | 3.90E-12 |
| rs187786174 | 1 | 2523811 | A | G | -0.117 | 3.30E-14 |  | rs1571878 | 6 | 167540842 | T | C | -0.151 | 6.10E-30 |
| rs2240339 | 1 | 17674108 | T | C | -0.128 | 2.50E-18 |  | rs1704997 | 6 | 33203908 | A | G | -0.288 | 3.40E-45 |
| rs2317230 | 1 | 157674997 | T | G | 0.077 | 2.10E-08 |  | rs17264332 | 6 | 138005515 | A | G | -0.163 | 6.70E-19 |
| rs2476601 | 1 | 114377568 | A | G | 0.593 | 1.00E-149 |  | rs17576984 | 6 | 32212985 | T | C | -0.431 | 3.00E-72 |
| rs28411352 | 1 | 38278579 | T | C | 0.113 | 3.60E-12 |  | rs1778478 | 6 | 28247638 | A | T | 0.182 | 1.30E-22 |
| rs72685699 | 1 | 113871830 | T | C | -0.211 | 4.10E-18 |  | rs17875360 | 6 | 30455321 | T | C | 0.113 | 7.20E-09 |
| rs761426 | 1 | 17413899 | A | T | -0.105 | 1.90E-09 |  | rs181997 | 6 | 32900718 | A | G | -0.174 | 5.10E-31 |
| rs10175798 | 2 | 30449594 | A | G | 0.086 | 5.40E-09 |  | rs1885205 | 6 | 36350992 | A | G | 0.140 | 1.60E-09 |
| rs11889341 | 2 | 191943742 | T | C | 0.131 | 6.70E-19 |  | rs206776 | 6 | 32953711 | T | C | -0.186 | 5.10E-37 |
| rs1858036 | 2 | 65598241 | A | G | 0.113 | 1.20E-14 |  | rs2228396 | 6 | 32797809 | T | C | 0.565 | 6.70E-166 |
| rs1980422 | 2 | 204610396 | T | C | -0.117 | 6.40E-12 |  | rs2239803 | 6 | 32411833 | T | C | 0.464 | 1.00E-250 |
| rs3087243 | 2 | 204738919 | A | G | -0.139 | 1.70E-22 |  | rs2485363 | 6 | 159506121 | T | G | -0.105 | 2.00E-10 |
| rs34695944 | 2 | 61124850 | T | C | -0.117 | 2.60E-13 |  | rs2516645 | 6 | 30501601 | G | C | -0.163 | 5.60E-09 |
| rs9653442 | 2 | 100825367 | T | C | -0.105 | 9.90E-15 |  | rs2517554 | 6 | 30993499 | T | C | 0.113 | 3.40E-16 |
| rs3806624 | 3 | 27764623 | A | G | -0.083 | 1.90E-08 |  | rs2523627 | 6 | 31346445 | T | G | 0.278 | 9.70E-85 |
| rs5019428 | 3 | 17046866 | A | G | 0.086 | 7.20E-10 |  | rs2523677 | 6 | 31434801 | T | C | 0.307 | 2.40E-37 |
| rs73081554 | 3 | 58302935 | T | C | 0.166 | 4.60E-08 |  | rs2596534 | 6 | 31445345 | T | C | -0.139 | 1.30E-24 |
| rs11933540 | 4 | 26120001 | T | C | -0.139 | 8.80E-17 |  | rs28381667 | 6 | 28783735 | A | G | -0.198 | 5.70E-15 |
| rs7683890 | 4 | 10726125 | T | C | -0.094 | 1.60E-08 |  | rs2854027 | 6 | 33180045 | T | C | -0.248 | 1.20E-40 |
| rs2561477 | 5 | 102608924 | A | G | -0.083 | 1.90E-09 |  | rs2856822 | 6 | 33047432 | A | C | 0.307 | 1.10E-99 |
| rs7731626 | 5 | 55444683 | A | G | -0.186 | 7.30E-24 |  | rs28744296 | 6 | 33440416 | T | C | 0.300 | 8.80E-34 |
| 6:30624155 | 6 | 30624155 | T | C | 0.140 | 9.20E-09 |  | rs28752520 | 6 | 32584739 | T | C | 0.693 | 7.70E-183 |
| 6:30661249 | 6 | 30661249 | A | C | 0.300 | 3.50E-32 |  | rs3131063 | 6 | 30763756 | A | G | 0.095 | 3.50E-13 |
| 6:31378228 | 6 | 31378228 | T | C | -0.288 | 1.40E-60 |  | rs35101948 | 6 | 30959417 | T | G | 0.392 | 5.80E-75 |
| 6:32007624 | 6 | 32007624 | A | G | 0.223 | 5.80E-18 |  | rs3857549 | 6 | 26406053 | T | C | -0.186 | 4.10E-10 |
| rs10484564 | 6 | 32752049 | G | C | 0.385 | 6.40E-29 |  | rs3869129 | 6 | 31410649 | T | C | -0.236 | 6.40E-37 |
| rs1079145 | 6 | 167360724 | A | G | -0.117 | 3.10E-09 |  | rs404860 | 6 | 32184345 | T | C | -0.151 | 7.60E-21 |
| rs112789728 | 6 | 32846335 | T | C | 0.207 | 1.50E-13 |  | rs41282672 | 6 | 44279309 | T | C | 0.104 | 4.90E-10 |
| rs113119046 | 6 | 33021130 | A | C | -0.416 | 1.10E-27 |  | rs423639 | 6 | 32987774 | T | C | -0.261 | 1.20E-20 |
| rs113239947 | 6 | 31461956 | A | T | -0.261 | 4.40E-52 |  | rs4360170 | 6 | 31430359 | A | G | 0.344 | 3.70E-23 |
| rs114139558 | 6 | 32749850 | G | C | -0.301 | 1.50E-16 |  | rs453779 | 6 | 32975381 | A | G | -0.198 | 1.20E-46 |
| rs4711325 | 6 | 33432709 | A | G | 0.086 | 8.60E-10 |  | rs9357094 | 6 | 30167476 | T | C | -0.248 | 2.00E-60 |
| rs4711363 | 6 | 33852948 | A | G | 0.122 | 7.70E-14 |  | rs9357165 | 6 | 33778619 | A | G | -0.139 | 2.80E-23 |
| rs4713424 | 6 | 31002742 | A | G | -0.211 | 6.90E-14 |  | rs9469556 | 6 | 33648661 | A | G | 0.215 | 8.30E-50 |
| rs55774724 | 6 | 33589332 | T | C | 0.131 | 3.00E-08 |  | rs3778753 | 7 | 128580042 | A | G | -0.105 | 1.10E-14 |
| rs59377618 | 6 | 32788137 | T | C | -0.223 | 1.90E-34 |  | rs2736337 | 8 | 11341880 | T | C | -0.105 | 4.80E-12 |
| rs60036207 | 6 | 32370794 | T | C | -0.528 | 1.30E-87 |  | rs11574914 | 9 | 34710338 | A | G | 0.113 | 2.10E-13 |
| rs6457718 | 6 | 33103827 | A | G | 0.140 | 6.40E-25 |  | rs1953126 | 9 | 123640500 | T | C | 0.086 | 1.00E-09 |
| rs671703 | 6 | 138147365 | A | G | 0.140 | 4.80E-12 |  | rs706778 | 10 | 6098949 | T | C | 0.086 | 1.50E-10 |
| rs68191 | 6 | 33480738 | T | C | 0.278 | 2.50E-25 |  | rs71508903 | 10 | 63779871 | T | C | 0.157 | 2.30E-20 |
| rs6906721 | 6 | 31270047 | A | G | -0.139 | 3.20E-15 |  | rs947474 | 10 | 6390450 | A | G | 0.104 | 1.50E-08 |
| rs6913550 | 6 | 26540683 | T | C | 0.104 | 8.70E-10 |  | rs11217002 | 11 | 118607150 | T | C | -0.128 | 6.80E-15 |
| rs6913635 | 6 | 33118937 | T | G | -0.416 | 8.40E-42 |  | rs4409785 | 11 | 95311422 | T | C | -0.105 | 3.00E-08 |
| rs6923504 | 6 | 32428186 | G | C | -0.616 | 1.00E-250 |  | rs61432431 | 11 | 128322622 | T | C | -0.094 | 3.60E-08 |
| rs6930468 | 6 | 426268 | A | G | -0.094 | 5.50E-11 |  | rs73013527 | 11 | 128496952 | T | C | -0.094 | 9.80E-11 |
| rs73418523 | 6 | 33861881 | A | C | 0.199 | 2.20E-10 |  | rs705699 | 12 | 56384804 | A | G | -0.083 | 6.50E-10 |
| rs7739491 | 6 | 30979203 | A | C | 0.166 | 1.50E-29 |  | rs9603616 | 13 | 40368069 | T | C | -0.105 | 4.60E-12 |
| rs7742523 | 6 | 29697898 | A | G | -0.128 | 5.30E-13 |  | rs168962 | 14 | 69282711 | G | C | -0.083 | 1.70E-08 |
| rs7772949 | 6 | 44259780 | G | C | -0.094 | 3.80E-08 |  | rs3784099 | 14 | 68749927 | A | G | -0.094 | 7.10E-10 |
| rs80281092 | 6 | 31455743 | A | G | 0.293 | 4.90E-30 |  | rs71411040 | 15 | 70001013 | T | C | 0.182 | 2.90E-12 |
| rs812561 | 6 | 31676641 | T | C | 0.300 | 1.20E-106 |  | rs8026898 | 15 | 69991417 | A | G | 0.148 | 6.50E-19 |
| rs9260532 | 6 | 29921235 | T | C | -0.211 | 4.40E-19 |  | rs8032939 | 15 | 38834033 | T | C | -0.117 | 4.80E-16 |
| rs9267548 | 6 | 31675985 | T | C | -0.400 | 1.60E-50 |  | rs13330176 | 16 | 86019087 | A | T | 0.113 | 1.60E-11 |
| rs9267655 | 6 | 31841055 | A | G | -0.139 | 5.90E-12 |  | rs59716545 | 17 | 38031857 | T | G | -0.094 | 1.20E-12 |
| rs9268541 | 6 | 32384527 | T | C | 0.519 | 7.50E-56 |  | rs487273 | 18 | 12853458 | T | G | 0.131 | 1.60E-15 |
| rs9271300 | 6 | 32581582 | G | C | -0.371 | 6.40E-114 |  | rs2304256 | 19 | 10475652 | A | C | -0.083 | 1.30E-08 |
| rs9271489 | 6 | 32589051 | A | C | -0.654 | 1.00E-250 |  | rs4239702 | 20 | 44749251 | T | C | -0.117 | 9.00E-15 |
| rs9272068 | 6 | 32599404 | A | C | 0.322 | 3.40E-46 |  | rs1893592 | 21 | 43855067 | A | C | 0.104 | 3.70E-12 |
| rs9296009 | 6 | 32114515 | A | T | -0.528 | 1.00E-250 |  | rs8133843 | 21 | 36738242 | A | G | 0.086 | 1.30E-09 |
| rs9296068 | 6 | 32988695 | T | G | 0.140 | 1.10E-22 |  | rs909685 | 22 | 39747671 | A | T | 0.113 | 6.30E-14 |
| **MS** | International Multiple Sclerosis Genetics, C. et al. Genetic risk and a primary role for cell-mediated immune mechanisms in multiple sclerosis. Nature 476, 214-219, doi:10.1038/nature10251 (2011) | | | | | | | | | | | | | |
| **SNP ID** | **Ch** | **Pos** | **EA** | **OA** | **Beta** | **P-value** |  | **SNP ID** | **Ch** | **Pos** | **EA** | **OA** | **Beta** | **P-value** |
| rs11581062 | 1 | 101407519 | G | A | 0.125 | 3.75E-10 |  | rs2844498 | 6 | 31476854 | T | C | -0.269 | 1.58E-54 |
| rs12746893 | 1 | 93210876 | C | T | 0.141 | 4.18E-12 |  | rs2894046 | 6 | 30782105 | C | T | -0.116 | 3.22E-10 |
| rs1335532 | 1 | 117100957 | G | A | -0.163 | 2.01E-09 |  | rs3095250 | 6 | 31208340 | C | T | 0.245 | 3.29E-47 |
| rs4648356 | 1 | 2709164 | A | C | -0.149 | 3.10E-14 |  | rs3129934 | 6 | 32336187 | T | C | 0.835 | 0 |
| rs11129295 | 3 | 27788780 | T | C | 0.109 | 1.14E-08 |  | rs3130559 | 6 | 31097301 | T | C | -0.195 | 1.70E-20 |
| rs2293370 | 3 | 119219934 | A | G | -0.150 | 1.08E-09 |  | rs3130604 | 6 | 32985052 | G | A | 0.237 | 4.72E-25 |
| rs2715267 | 3 | 121770858 | G | T | 0.108 | 4.17E-09 |  | rs3763309 | 6 | 32375973 | A | C | -0.318 | 2.61E-49 |
| rs4285028 | 3 | 121660664 | C | A | -0.113 | 3.71E-08 |  | rs3763349 | 6 | 32808232 | A | G | 0.199 | 1.29E-31 |
| rs669607 | 3 | 28071444 | C | A | 0.121 | 2.93E-11 |  | rs385306 | 6 | 31681160 | A | G | -0.133 | 3.18E-11 |
| rs771767 | 3 | 101748638 | A | G | 0.113 | 1.00E-08 |  | rs404860 | 6 | 32184345 | C | T | -0.310 | 1.87E-37 |
| rs9282641 | 3 | 121796768 | A | G | -0.191 | 1.54E-09 |  | rs4713610 | 6 | 33107955 | G | T | -0.229 | 5.99E-22 |
| rs4613763 | 5 | 40392728 | C | T | 0.194 | 6.86E-14 |  | rs482759 | 6 | 32195017 | G | A | -0.217 | 4.97E-22 |
| rs1015166 | 6 | 32798731 | T | C | -0.140 | 8.43E-15 |  | rs719654 | 6 | 32752139 | A | G | -0.221 | 1.53E-26 |
| rs1041981 | 6 | 31540784 | A | C | -0.191 | 2.11E-26 |  | rs9277565 | 6 | 33056897 | T | C | 0.169 | 1.86E-16 |
| rs11154801 | 6 | 135739355 | A | C | 0.137 | 1.53E-12 |  | rs9295895 | 6 | 30438226 | C | T | -0.125 | 9.83E-10 |
| rs11757000 | 6 | 28484869 | C | T | 0.206 | 3.77E-18 |  | rs9378200 | 6 | 31572927 | C | T | -0.342 | 2.16E-20 |
| rs12210887 | 6 | 31815723 | T | G | -0.266 | 4.57E-12 |  | rs3118470 | 10 | 6101713 | C | T | 0.116 | 2.05E-09 |
| rs16896742 | 6 | 29922740 | G | A | -0.266 | 8.03E-52 |  | rs650258 | 11 | 60832282 | T | C | -0.112 | 1.74E-09 |
| rs17066096 | 6 | 137452908 | G | A | 0.128 | 3.42E-10 |  | rs1800693 | 12 | 6440009 | C | T | 0.111 | 1.84E-10 |
| rs1738074 | 6 | 159465977 | T | C | -0.124 | 5.27E-11 |  | rs2119704 | 14 | 88487689 | A | C | -0.233 | 3.46E-10 |
| rs17500468 | 6 | 32711178 | G | A | -0.197 | 2.73E-15 |  | rs4902647 | 14 | 69254191 | T | C | -0.103 | 3.81E-08 |
| rs17576984 | 6 | 32212985 | T | C | -0.194 | 8.52E-11 |  | rs7200786 | 16 | 11177801 | A | G | 0.143 | 6.30E-14 |
| rs206769 | 6 | 32961104 | T | C | -0.130 | 5.25E-10 |  | rs1077667 | 19 | 6668972 | T | C | -0.149 | 2.13E-12 |
| rs2517459 | 6 | 30897022 | T | C | 0.259 | 2.98E-39 |  | rs874628 | 19 | 18304700 | G | A | -0.114 | 4.32E-08 |
| rs2523589 | 6 | 31327334 | T | G | -0.247 | 1.01E-48 |  | rs2248359 | 20 | 52791518 | T | C | -0.112 | 5.15E-09 |
